# Facilitating and hindering factors of personal recovery in the context of Soteria—A qualitative study among people with (early episode) psychosis

**DOI:** 10.3389/fpsyt.2022.1051446

**Published:** 2023-01-04

**Authors:** Pien Leendertse, Fadi Hirzalla, David van den Berg, Stynke Castelein, Cornelis Lambert Mulder

**Affiliations:** ^1^Emergis, Institute for Mental Healthcare, Kloetinge, Netherlands; ^2^Department of Psychiatry, Erasmus University Medical Center, Rotterdam, Netherlands; ^3^Department of Public Administration and Sociology, Erasmus School of Social and Behavioural Sciences, Erasmus University, Rotterdam, Netherlands; ^4^Department of Clinical Psychology, VU University and Amsterdam Public Health Research Institute, Amsterdam, Netherlands; ^5^Research and Innovation, Parnassia Psychiatric Institute, The Hague, Netherlands; ^6^Lentis Research, Lentis Psychiatric Institute, Groningen, Netherlands; ^7^Faculty of Behavioral and Social Sciences, Clinical Psychology and Experimental Psychopathology, University of Groningen, Groningen, Netherlands; ^8^Antes, Institute for Mental Healthcare, Parnassia Psychiatric Institute, Rotterdam, Netherlands

**Keywords:** psychosis, Soteria, personal recovery, subjective recovery, inpatient treatment

## Abstract

**Objective:**

The objective of this study was to gain insight into patients' experiences of how personal recovery (PR) is facilitated or hindered in the context of an early episode psychosis setting (Soteria). We thereby aimed to contribute to the understanding of how care settings may promote or hinder the process of PR in people with (acute) psychosis.

**Method:**

This study used a qualitative method, consisting of semi-structured in-depth interviews with people who had been admitted to a Soteria house in the Netherlands. Interview transcripts were analyzed following the Grounded Theory approach.

**Results:**

Five themes emerged from the data illustrating how Soteria facilitated or impeded PR. The experience of togetherness in contact with staff and peers, feeling at home, and being active facilitated PR, while the emphasis put on medication by staff was experienced as hindering, and attention to spirituality was missed.

**Conclusion:**

In addition to the literature that identified factors associated with PR in psychosis, the current study gives a sense of how this can be put into practice. By offering treatment within a normalizing, holding environment, with emphasis on equality, close contact, optimism, active structured days, open-mindedness toward spirituality, and the role of medication, PR can be facilitated without detracting from guideline-based treatment aimed at symptomatic recovery. Similarities with existing concepts are discussed.

## 1. Introduction

Soteria is a small-scaled, homelike facility for people with an early episode of psychosis in need of hospitalization. Key ingredients are offering a warm, calming therapeutic environment, with normalizing days (e.g., three daily meals and a balance in activity and relaxation), spent within a group of patients and staff. A phenomenological approach toward psychosis is applied, aiming to give meaning to a person's subjective experience of psychosis and to come to a shared understanding of symptoms within the individual social context. In line with this, the staff is instructed that their main task is “to be with,” meaning that they are first of all expected to be present and stand beside the patient (1). Soteria originated from a research project in the 1970's (2), which aimed to counterbalance the then-prevailing hierarchical medical approach to psychosis. No or low-dose antipsychotic medications were prescribed, based on the assumption that the therapeutic milieu itself could be a sufficient agent for treating acute psychosis (1). In the years after, the model evolved toward a holistic framework, in which much attention is paid to psychological and social interventions, next to guideline-based low-dose medications (3, 4). To date, several Soteria houses and wards with Soteria elements are operating around the world (5, 6). Previous research into Soteria focused on clinical and functional outcomes. This research was summarized in a systematic review by Calton et al. (7), which included three controlled trials involving a total of 223 participants with a first or second episode of psychotic disorder. Their findings suggest that when compared to controls treated with conventional, mainly medication-based approaches, inpatient treatment of early episode psychosis in Soteria yields equal clinical recovery and better results in certain areas of functional recovery (e.g., independent living and occupational functioning). However, since Soteria's focus is on a person's subjective experience of psychosis, it would be interesting to explore—from a patient perspective—whether and how Soteria contributes to a more subjective form of recovery.

Recovery is a multi-dimensional concept. Clinicians tend to describe recovery as the reduction of symptoms and improvement of functioning (symptomatic or clinical recovery), while patients may experience recovery to be broader than or separate from symptoms or functioning. The patients' perspective on recovery is a subjective and highly personal concept and is often referred to as “personal recovery” (PR) or subjective recovery. A large and growing body of literature on the conceptualization of PR exists, on the basis of both qualitative and quantitative research. However, a widely endorsed conceptual framework for PR in severe mental illness is offered by Leamy et al. (8), which revolves around connectedness, hope, identity, meaning in life, and empowerment (CHIME). When it comes to PR in psychosis, to date, several literature reviews have been published. A thematic synthesis of qualitative studies from a patient perspective outlines social support, faith and spirituality, personal agency, and hope as facilitators of PR, and stigma and discrimination, negative effects of mental healthcare services, and medication as barriers to recovery (9). The review of Jose et al. (10) describes how PR in psychosis mainly involves factors related to personal wellbeing and social inclusion that were seemingly distant from clinical recovery measures. Indeed, reviews of quantitative nature suggest that personal and clinical recovery appear to be related, but separate constructs; people who still experience symptoms might nevertheless report PR (11, 12). Overall, the conceptualization of PR and its distinction from clinical recovery is becoming increasingly clear, and PR is becoming a popular outcome measure in clinical practice. Therefore, more research is needed into factors facilitating PR in specific populations (13). Examining what contributed to patients' process of PR could gain insight into potential active ingredients for PR-oriented interventions (12).

Soteria is believed to be a PR-oriented care setting, because of its phenomenological approach to psychosis and broader focus than the reduction of symptoms. There are several theories about how Soteria may contribute to recovery from psychosis. Ciompi's “theory of affect-logic” states that the environmental and social context of a Soteria house reduces affective tension and thereby promotes remission from symptoms (14). A recent essay hypothesizes that this affect-logic is induced by the ability of the Soteria approach to adapt to and restore self-disturbances, as a central element of psychosis (15). However, no studies have explored how patients themselves perceive the role of Soteria in recovering from psychosis. Therefore, the objective of this qualitative study is to explore how patients with psychosis experience the way in which Soteria facilitates or impedes PR. The overall aim is to contribute to the understanding of how a care setting may promote the process of PR in a population of people with psychotic disorders.

## 2. Materials and methods

### 2.1. Design

To gain insight into the patient's perspective on the role of Soteria in PR, we used a qualitative method, consisting of semi-structured in-depth interviews with people who had been admitted to the only operating Soteria house in the Netherlands (Soteria NL). Our inductive approach enabled us to explore how participants conceptualize their experiences and the role of Soteria in PR in their own words, which forms an addition to the findings of the more deductive research performed, thus, far. At the moment of the interview, participants were discharged from Soteria and only received outpatient care. Anonymity was emphasized, as well as the fact that given responses would have no consequences for the current treatment. We received an exception from further review by the Medical Ethical Committee as the current study was considered as not being subject to the Dutch Medical Research Involving Human Subjects Act (MEC-2021-0152).

### 2.2. Setting

Soteria NL is an inpatient setting that can accommodate 10 people in need of voluntary or forced hospitalization, with an acute episode of a psychotic disorder (e.g., brief psychotic disorder, bipolar-I disorder, and psychotic disorder not otherwise specified). Patients are mostly aged 16–35 years. Soteria is part of a regular mental healthcare service located in Kloetinge, in the southwest of the Netherlands. People are referred to Soteria NL either directly by the crisis service, general (closed door) admission wards, or ambulatory care (FACT teams). Clear and concordant information on psychosis, treatment goals, and selection of interventions are discussed with the patients and their families at the start of the stay. Progress is evaluated every 4 weeks in meetings with the patient and others involved. These meetings are chaired by the patient, to increase empowerment and self-determination. Both family and the ambulatory team are actively involved in treatment from beginning to end, to ensure personal continuity and thereby facilitate the transition during discharge. The duration of stay in Soteria NL ranges from several weeks to almost a year. The search for appropriate housing or daytime activities after leaving Soteria is the most common reason for longer durations of stay. The staff consists of a small team of nurses and social workers, working in 12-h shifts offering continuous personal “being with” the person with psychosis, together with experts by experience, volunteers, and a part-time psychiatrist and psychologist. Treatment takes place in a normalizing, homely decorated setting. There is a structured program of day-to-day activities, which are organized outside of mental healthcare (e.g., at the local gym) and attended by both patients and staff. Treatment is guideline-based, within which emphasis is placed on group conversations, social recovery goals (housing, social network, daytime activities), involvement of relatives, psychological treatment (cognitive behavioral therapy for psychosis, trauma treatment), and pharmacological treatment. An overall description of Soteria and its theoretical foundations and aims can be found in Ciompi and Hoffman (16).

### 2.3. Inclusion and recruitment of respondents

Sampling was purposefully done *via* case managers of patients that had been admitted to Soteria in the past and were now receiving community treatment. We interviewed patients who were at least 18 years old and capable of having an hour-long conversation with us. We only interviewed patients who stayed longer than 4 weeks at Soteria. We approached all patients admitted to Soteria in a given year who met the inclusion criteria. In this way, we attempted to avoid selection bias. The specific year in which respondents were recruited is not provided to prevent the combination of indirect identifiers (e.g., age, gender, diagnosis, and year of admission) that would lead to identifiable persons. We interviewed as many respondents as was necessary to reach data saturation, meaning that conducting more interviews would not provide new insights to accomplish our research goal.

### 2.4. Data collection

After having provided written informed consent, the interviews took place at a location of choice of the participants (mostly at their homes) in 2021. We started with general questions that explored what PR meant to the respondents and whether and how Soteria facilitated or impeded their PR. We asked respondents, for instance, “what recovery means” to them and “what role Soteria played” in their recovery. More specific conversation topics (such as symptoms, medication, identity, hope, cognitive and social functioning, and spirituality) were derived from the participants' initial responses or the relevant literature [e.g., (17–19)]. Keeping our approach inductive, we explored literature topics through open questions about their relevance and meaning for the respondents in relation to their PR. For example, regarding the topic of hope: “What role did hope play in your recovery?” and “In which way was attention given to this at Soteria?”. Followed by: “How was this helpful/ hindering?”. In the end, we asked the respondents whether they have any suggestions for how Soteria could improve their service, and we checked whether there was anything else they liked to add to the conversation.

Two pilot interviews were conducted with admitted Soteria patients. Their input led to displacing questions about the meaning of PR to the start of the interviews, in order to better understand the context of the answers that followed after. It also led to deleting questions about the meaning of psychosis, in order to limit the conversations to an acceptable duration of an hour. Both interviews were not included in the data analysis.

The topic list was also determined through theoretical sampling. In grounded theory, theoretical sampling aims to attune later steps in the data collection process to preliminary findings from earlier steps in the data collection (20). After each interview, memos were taken and discussed within the review team on the basis of which the next interview was pursued. After seven interviews, the process of coding started leading to decisions on what data to collect next, based on the theory emerging from the data. The final evaluation protocol can be found in the Supplementary material.

The interviews were tape-recorded, transcribed verbatim, and anonymized. The first author (PL) conducted all 10 interviews. A psychology student (MD) participated as a research assistant in the first six interviews. PL had a prior relationship with the participants as their psychologist during their stay at Soteria. MD did not have this prior relationship, which helped broaden the variety of questions asked. A psychiatrist with experience in qualitative research (AR) served as a third member of the review team.

### 2.5. Data analysis

All interviews were coded by PL, MD co-coded interviews one through six separately, and AR co-coded interviews seven and eight. Memos and field notes were kept throughout the data collection process. Member checks were offered in all cases. A sample of the interviews was reviewed during data collection by AR, checking for omissions and possible misinterpretations. Interview transcripts were analyzed by two members of the research team (PL and AR) following the grounded theory approach (21). The constant comparative method was used, through which all pieces of data are compared to each other in order to discover patterns in the data. This process was facilitated by coding. Analysis was performed on the transcripts with the program Atlas.ti 9. Quotes of participants in this article are translated into English. After a process of open coding, aimed at describing the data on a specific level with substantive labels, the analysis continued with axial coding, which entailed comparing codes to each other and discovering combinations of and relations between codes. Emerging concepts and categories were shared between PL and AR and discussed along with memos about observations and insights. The researchers kept notes of their discussions of the process during the analysis. They continually searched for a shared understanding of the codes as they were developed. Finally, through the process of selective coding, PL and AR assessed whether and how the data could be coded further in relation to the main themes (22). Through these three steps, we were able to discover how our respondents conceptualize PR and perceive Soteria's role in PR. Our core findings are reported in the next section on the basis of the COREQ guidelines (23).

## 3. Results

After interviewing 10 respondents, the saturation of the data was reached. At this point, the coding process indicated that assessing more data does not yield any substantial new insights. Seven respondents were male subjects, the rest were female subjects. The respondents ranged in age from 21 to 42 years (the mean age was 27.9 years). The duration of stay at Soteria in our sample ranged from 2 to 10.5 months. No indications of a relationship were found between given responses and length of stay. Patient characteristics are shown in Table 1.

**Table 1:** Patient characteristics.

**Respondent**	**Sex**	**Age**	**Classification (DSM-IV or V)**	**GAF score**	**Duration of stay (months)**
1	M	20–25	Brief psychotic disorder	40	2
2	M	25–30	Unspecified catatonic disorder	45	6
3	F	30–35	Brief psychotic disorder	40	4.5
4	F	30–35	Bipolar I disorder	45	6
5	F	20–25	Bipolar I disorder	45	9.5
6	M	20–25	Bipolar I disorder	40	5
7	M	30–35	Brief psychotic disorder	30	10.5
8	M	25–30	Brief psychotic disorder	45	2.5
9	M	25–30	Psychotic disorder NOS	45	2
10	M	40–45	Schizophrenia undifferentiated type	50	3

M, male; F, female; DSM, diagnostic and statistical manual of mental disorders; version IV or V; GAF, Global Assessment of Functioning.

Personal recovery was conceptualized by the respondents in different ways. For instance, from the disappearance of—or the ability to accept—symptoms to regaining previous roles and levels of functioning, or mainly a personal process of slowing down or feeling good about yourself again. Some respondents referred to PR as a largely autonomous process, while others saw it more as an outcome that was reached in cooperation with others. However, regardless of these variations in conceptualizing PR, respondents could give clear and rather uniform descriptions of what had been helpful or hindering for their PR during their stay at Soteria. Below, we will discuss how five themes emerged from our data that illustrate how Soteria facilitated or impeded these diverse and sometimes general descriptions of PR from psychosis.

The first theme, *togetherness*, describes how PR is facilitated by the support that is experienced in the nature of contact with staff and peers. The second theme, *feeling at home*, is about the helping role of the homelike setting and atmosphere. The third theme, *being active*, illustrates how structured days and normalizing and creative activities can facilitate, and sometimes hinder PR.

These first three themes were mentioned spontaneously by respondents when asked about the role of Soteria. The fourth and fifth themes emerged from responses to topics initiated by the interviewers. The fourth theme pertains to spirituality, which might play a major facilitating role in PR, but was largely experienced not to be an integral part of care. The fifth theme centers on the role of medication. While it became clear that medication can both help and hinder PR, respondents noticeably hesitated to speak openly about this topic. The themes will be described one by one in the text below, and illustrated by anonymized quotations.

### 3.1. Theme 1: Togetherness

#### 3.1.1. Nature of contact between patients and staff

According to the Soteria principles, the group of patients and staff functions as a community or large family in which the nature of contact is characterized by non-hierarchical relations and staff continuously “being with” patients. Respondents spoke passionately about the nature of contact with staff members, which they generally characterized as familiar, trusted, and close. In part, this was facilitated by the fact that the team of staff members was relatively small with little turnover. Respondents mentioned that conversations with staff members and other patients were about everything that mattered to them in life in general, rather than only about (psychotic) symptoms. The fixed and long-term relations between patients and staff and the candid conversations about life at large made respondents experience the community at their treatment facility as a “family” that they could trust, to whom they stood close, and that supported them as “persons” rather than mere “patients.” This is illustrated by excerpts 1 and 2.

*1: For me it's like, when I enter Soteria, it's like I'm returning to family… You build a bit of a relationship with staff there… I also think it's just, the way they are. They're open and not afraid to reveal themselves… especially when you're not feeling great or you're psychotic, than trusting others, uhh, well maybe you're suspicious or something. So trusting others might be tough… For me it's always pleasant at Soteria to know that I can trust everyone there… (Respondent 1, male, 20–25 years old)*.

*2: They're engaged [Staff]. They're very engaged. And you don't need to… you are taken seriously. They see you as a person, so to speak, as who you are… They were sincerely interested in where you are in life, and also willing to think along. You could always count on them, and also speak about different topics… Those kind of conversations were valuable… (Respondent 4: female, 30–35 years old)*.

Although respondents had difficulty explicating how this nature of contact with staff contributed to PR, all respondents emphasized that these were the elements that had helped them most during their stay at Soteria. Perhaps familiar and close contact and being supported as a “person” rather than a mere “patient” could be perceived as “enabling conditions” to attain, for example, the reduction of symptoms of paranoia, or finding a new perspective in life as a person prone to psychosis instead of a patient.

#### 3.1.2. Peer-to-peer contact

Having frequent and close contact with other early-episode psychosis patients was also important to the respondents. Most respondents experienced spending time with others and sharing thoughts and feelings about psychosis as pleasant and supportive. Such peer-to-peer contact gave a feeling of “being in there together,” in the words of one respondent. Soteria's social environment seems to foster these feelings of togetherness by encouraging patients to spend most time and do all sorts of activities together. This is illustrated by respondent 3:

*3: Actually, by doing all these activities together… And that you're actually going through this recovery process together. That is really much more pleasant than having to do everything alone… (Respondent 3, female, 30–35 years old)*.

The group discussions enabled the respondents to learn about psychosis in general and about the experiences with psychosis of other patients in the group. The knowledge the respondents gained in this process, they said, supported their recovery. It helped, for instance, with “situating” their struggle of finding words or the explanation for their psychotic experiences, in the words of respondent 4:

*4: You just have more knowledge about psychosis, so you are better able to picture the things you are dealing with, I think… Things that have happened in the past, or also, more knowledge about what psychosis is, how you could explain it to others, or how you can deal with the ignorance of people around you*… (*Respondent 4, female, 30–35 years old)*.

The group discussions also familiarized the respondents with talking openly about their psychotic symptoms. Respondent 10, for instance, mentioned that he talked “*about it easier now.. others have these experiences too, I know that now”* (respondent 10). Respondent 3 described the added value of learning about psychosis and concurrently sharing experiences for PR as follows:

5: *It really provided a lot of insight, but also a piece of connection with people, that you're not alone. And this really has added value for me I think, and also for my recovery, because you know that you are not alone, and that more people are going through this… (Respondent 3, female, 30–35 years old)*.

Naturally, spending your days in intensive contact with others- this accounts for people in general, and for people going through a recovery process in specific - also brings along having to be considerate of each other. Therefore, some respondents described the intensive peer-to-peer contact as hindering. For instance, one respondent had difficulty with group members not meeting commitments. “*Actually, I mostly remember that I was annoyed by the rest showing up too late, or not at all, or those who were always first*” (respondent 8). Another respondent mentioned that the intensive group setting made him feel unsafe: “*And then security came running down the hall, and then yes, that was scary for a while. At that time I did feel a tiny bit unsafe”* (respondent 9). Noticeably, these respondents reported that these nuisances did not outweigh the benefits of “being in there together” for their recovery.

### 3.2. Theme 2: Feeling at home

A second theme that respondents spoke passionately about was the homelike setting of their facility. Homeliness was in part induced by the architecture and design of the treatment center, which respondent 4 described as “cozily furnished” (excerpt 6). Homeliness was also created by the fact that patients and staff “lived together” in the facility. For instance, they shared their meals and the responsibility for the household, which sustained the close ways of interaction and communication within the group. The physical and social setting of the facility gave respondents the feeling that Soteria is like a home for them where residents shared, as respondent 9 described it (excerpt 7), a “sense of community.”

*6: It was just homely there. And it made me feel good, also when I look back. Every time I get to [city] I think about Soteria (smiling) and then I also think about the good things, so to speak… It was in the way we interacted with each other. But also in the furnishing. For instance, there was a specially furnished living room. It was cozily furnished. And that also contributed to the homelike feeling, the perception, the experience… (Respondent 4, female, 30–35 years old)*.

*7: It is a kind of homelike atmosphere… you eat together, you do all kind of activities together… It is in the arrangement of furniture… It causes a sort of community feeling, and that is kind of what it's all about… Eventually it is all about having a nice time, and coziness is very important in this I think, because you get to know each other in a different way… (Respondent 9, male, 25–30 years old)*.

Interestingly, all respondents looked back on their stay at Soteria in a pleasant way, despite the fact that being hospitalized was a difficult period in their life. Respondent 4, for instance, described her stay as “*a nice memory… a hard time but still a nice memory.”* Respondents also stressed that it was helpful that there was room for coziness and laughter, and that they could feel an optimistic or cheerful atmosphere. This finding is also relevant in light of the relatively high risk of relapse and readmission rates in the early years of psychotic disorders. Taken together, the homelike and familiar setting and the nature of contact between patients and staff, within an optimistic and cheerful atmosphere, appears to promote PR by making people feel at home. Feeling at home appears to create space for a process of “getting back to normal” again.

### 3.3. Theme 3: Being active

At Soteria, days are structured around the three daily meals, shared responsibility of the household, and a range of creative and physical activities organized outside of mental healthcare (e.g., the local gym, arts, music, yoga) alternated with group conversations. All respondents described the activities as a distinctive feature of their stay. Most respondents mentioned that being active facilitated PR, but it also hindered PR among others.

Being active first promoted PR by creating a distraction from symptoms, which respondent 4 described as “*not being constantly concerned with your own problems*” (excerpt 8). Respondents believed distraction was important to mitigate their suffering: when there is no distraction, suffering might worsen. “*For me*,” respondent 1 said, “*it was disastrous to do nothing all day when I suffered from my voices, then they'll only get worse.”* Being active in structured days also helped with regaining previous functioning by what was described as creating “*challenges*,” and respondents mentioned that it provided a feeling of contributing, of being “*sort of needed*” (excerpt 9). Respondent 1 illustrated this by saying “*something is expected from you as well.”*

*8: I think by being active, you can get your mind off of things. You are not constantly dealing with your own problems. And I think it is also helpful that you are being busy with other, daily stuff… You do not constantly have to think about your psychosis, so to speak… Also, some structure and tranquility is good for you as well… (Respondent 4, female, 30–35 years old)*.

*9: You were also required to cook, for example. And required to participate in a program, that actually consisted of all kinds of daily activities. So you were really taken along… You recover faster compared to a situation where there are no challenges at all… It makes you feel like you still matter in society really. Because you feel like sort of needed, or, at least that you also have a task to accomplish. Yes, that just gives satisfaction… (Respondent 3, female, 30-35 years old)*.

However, as opposed to the distracting and activating role of structured days, some respondents experienced constantly being busy as a burden. For instance, the activities made some respondents feel incompetent. This is illustrated by respondent 4, who said “*I felt burdened… or a bit of, oh, I can't do it, it's very hard to motivate myself…”*.

Respondents also emphasized the nature of the activities pursued. Being active in “*small, regular activities*” as described by respondent 5 (excerpt 10 below), like household chores in and around the house, were described as aiding their PR by making them “*feel normal*”. Participating in these activities appears to place less emphasis on the role of the “patient” and more, as respondent 5 described it, on “*the human part*”. Next to small regular day-to-day activities, creative, non-verbal, or physical activities—like arts or sports—promoted PR by creating physical relaxation and helping with “*connecting to your own body*”, in the words of respondent 3 (excerpt 11).

*10: For example, doing really small things together, just regular activities, because than you're doing something… It is better anyway, for someone with psychosis, or depression, or mania, not to do nothing. Because than you just ‘stop the machine'. You could compare it with a car; you should not let it stand still for too long, you just have to keep going. Okay, not in full speed, but.. It is important that the focus is put on something else for a moment. On the human part… (Respondent 5, female, 20–25 years old)*.

*11: The horse-riding really contributed to a fast recovery, because you have to keep balance on a horse. And also yoga, because it helps you connect to your own body. And that's exactly what the problem is in a psychosis, for me it was the feeling that I was really far out and not in my body. With yoga and also the horse-riding, you just had to be in your body… (Respondent 3, female, 30–35 years old)*.

### 3.4. Theme 4: Spirituality

Respondents were asked to reflect on the role of spirituality in their PR. The term spirituality is admittedly vague and broad. We used it in the interviews as a proxy to find out if and how respondents found solace in empowering thoughts and ideas, based on the assumption that a person, for instance, can find strength in religion, or that a spiritual explanation of symptoms might provide a better grip on a situation. However, we kept the definition of spirituality open, and only offered examples of spirituality if needed for respondents to reflect on this. Accordingly, respondents mentioned different forms of spirituality, ranging from the belief in God or “*something bigger*” to meditation or prayer. For some respondents, spirituality plays no part in PR at all. “*We did go to yoga*,” respondent 9 said, “*and I do believe there is a heaven.. but in relation to recovery, no…*”. Others said that spirituality played a major role in PR. Respondent 1, for instance, described it as inducing “*radical change*” (refer to excerpt 12), and respondent 4 described religion as the “*the key*” factor in reaching PR: “*It is really my key… It just brings you strength, hope, a future. And you're being confirmed in who you are… I couldn't understand to go through this without my religion, I just couldn't.*…”

*12: Religion and spirituality helped me a lot, because I just realized that I had lost myself. And I did not love myself, and I came to realize that I was really frustrated with myself, and that this had to change. And then I started to learn about God, spirituality, meditation. And then, uhh, there was a radical change, from there it only got better and better… (Respondent 1, male, 20–25 years old)*.

Although the importance of spirituality for PR was stressed by some respondents, it was not experienced as part of the treatment that the respondents received, neither in group conversations nor in individual therapy. Perhaps this explains in part why respondents did not mention spirituality spontaneously as a facilitator in reaching PR.

When respondents were asked whether they had missed attention to spirituality, some respondents answered negatively. For instance, respondent 7 said: “*I think there would probably be room for that, but I don't feel the need to have a conversation about it, I just don't need that…”*. Some respondents confirmed having missed room for spirituality, and—in a broader sense—meaningfulness, or even described it as an “*underappreciated topic*” (excerpt 13).

*13: Spirituality is a highly underappreciated topic, because in a psychosis all these crazy things pass by. It seems like some kind of alternate reality, and first I was really afraid of these things, but nowadays I can say that I have actually learned a lot from it. I can deal with these things now, and I also believe that disease has a special meaning in your life. Actually, now speaking about it, I have really missed this spiritual aspect of what meaning you give to disease or illness, but also about the content of psychotic symptoms for example. I really would have liked to hear from others what they experienced in their psychosis for example, are there similarities? I would have found that really interesting… (Respondent 3, female, 30–35 years old)*.

### 3.5. Theme 5: Medication

One of the distinguishing elements of Soteria compared to the usual (inpatient) treatment of acute psychosis is the primary focus on the therapeutic milieu, instead of medication. The role of medication in PR varied considerably. For some respondents, medication merely facilitated PR. For instance, respondent 1 said: “*Yes, medication mainly brought relief. It made things bearable*.” However, the majority of respondents had “*mixed feelings*” about medication, describing it as something that offered both “*relief* ” and “*burden*” (see excerpts 14 and 15). Finally, some respondents described the medication as neither hindering nor facilitating PR. For instance, respondent 10 said: “*It's not a negative effect and I don't notice a positive effect either. I don't notice any difference….”*

*14: Yes, they say it's very important. Yes, no, I do also believe that, you know. I do think that medication is, it's, uhh, to calm you down as soon as possible, and get out of that psychotic state. That is just really important… I just wish I didn't need them. On the other hand, I should be happy that medication is available. It is a bit of a mixed feeling, so to say…” (Respondent 4, female, 30–35 years old)*.

*15: When you no longer experience symptoms, and you still take medication, you literally feel muted in everything that you do. You just get blinded. So on a certain point, I just became so, actually, sort of, very burdened by these medication. I just don't feel good about that. But that realization only came after the voices disappeared, because before, medication was more of a relief. And now it's more of the other way around… (Respondent 1, male, 20–25 years old)*.

Considering the perspective on medication within the Soteria paradigm, another interesting finding is that the majority of respondents felt that medication was “*prominently present*” (respondent 2) and “*considered very important*” (respondent 6) in treatment by staff. Because of this, taking medicine was experienced as “normal” by the respondents. It was “*part of the game*,” in the words of respondent 9, which could make patients even feel they have an obligation to take the medicine:

*16: Well that was quite a step for me actually. Uhm, at first, I mildly, uh, resented it, well, it was a bit unfamiliar. How would it affect me… I didn't really like the idea of medication actually, uh, initially. But yes, that was, uh, so I took them anyway, partly based on the insistence of my family, and also uh, well, then you're impressed with Soteria, and then you think, yes, that's part of the game here, so yes, so then I accepted the medication… Uhm, and if it was helpful? Uh.. well, uh… yes well, at a certain point you just don't know any better, so to speak… and uhm, uhm, was it helpful, yes, well, it doesn't bother me, so to speak, those medications… (Respondent 9, male, 25–30 years old)*.

Perhaps, in part, as a result of the emphasis on medication by staff, the respondents hesitated to speak about this topic. Some respondents only spoke about medication after it was introduced as a topic by the interviewers, and when we asked about it, some of the respondents gave vague answers with a lot of “*uhhs*,” or they started to talk in the third person. The emphasis on medication by staff might have hindered participants to speak openly about the potential negative effects of medication on PR altogether.

## 4. Discussion

The objective of this study was to explore how patients experienced the way in which Soteria facilitated or hindered their PR from psychosis. Five themes emerged from the data; togetherness (in contact with staff, and with peers), feeling at home, being active, spirituality, and medication.

The first three themes present facilitating factors. First, experiencing togetherness in familiar and close contact with staff and peers facilitates PR. This is in line with Mosher's idea of “being with” (1). It is also in line with several dimensions of the CHIME framework (8). For instance, the dimension “connectedness” describes the support of peers and social groups as well as the importance of relationships. Respondents in our study emphasized the importance of equal and normalizing contacts, in which they are seen as “persons” rather than “patients.” This is in line with the CHIME dimension “identity,” containing the aspects of rebuilding a positive sense of identity and overcoming stigma. Interestingly, respondents also emphasized the optimistic cheerful atmosphere, which is in line with hope-inspiring relationships and positive thinking described within the CHIME dimension “hope and optimism about the future.” This also led to predominantly positive feelings described by most respondents while looking back at their stay. This is relevant since relapse rates are relatively high in the early years of psychotic disorders (24), and inpatient admissions have often been experienced negatively, or even traumatizing, for people who experience psychosis (25).

The importance of normalization is illustrated by the fact that it is reflected in the first three themes; normalization is fostered through regular and close social contacts (theme 1), the homely setting at Soteria (theme 2), and daily leisure and household routines (theme 3). Respondents could of course be especially helped by such conditions if they previously led an abnormal lifestyle that sustained their psychosis. This appears to be in line with gaining a sense of control over life and personal responsibility, captured in the CHIME dimension “empowerment.” Furthermore, all three themes have in common the fact that they defy (self-)stigma, which is known to inhibit PR in young (hospitalized) people with psychosis (26, 27). The importance of stigma and social inclusion is also emphasized in reviews of qualitative research into PR from psychosis from a patient perspective (9, 10).

The last two themes present factors that were perceived to be mainly hindering. The fourth theme describes how spirituality was not explicitly integrated into care, while it appears to be an important facilitator of PR, at least for some respondents. Finding meaning in psychotic experiences, as described by Feyaerts et al. (28) would be well in line with the phenomenological approach to psychosis practiced in Soteria houses. Furthermore, the importance of open communication about causal beliefs of psychosis between patients and staff is emphasized in a recent scoping review (29). Since not all respondents mentioned spirituality as important for their PR, and spirituality might be conceptualized differently among patients and professionals (30), the collaborative exploration of meaning or spiritual dimensions of psychosis is important. This might have been a facilitator of PR that was left unused.

Finally, the fifth theme describes how respondents experienced that staff emphasized the importance of medication, which might have hindered respondents to speak openly about medication and potentially hindering side effects. This finding was unexpected since medication is explicitly not the primary intervention according to the therapeutic principles of Soteria (16). It is also unexpected in light of the first theme in which contact with staff was described as close and familiar. The fact that medication was described by the majority of respondents as “prominently present in treatment” might have had to do with the fact that our Soteria house was organized within a mental healthcare institute. A review of qualitative studies describes (side effects of) medication as a barrier in reaching PR (9).

Overall, in his “theory of affect-logic,” Ciompi hypothesized that the environmental and social context of a Soteria house reduces affective tension, and thereby promotes remission from symptoms (16). Our results add to this theory by suggesting that the environmental and social context facilitates PR by inducing feelings of inclusion and defying stigma. This latches on research emphasizing the importance of environmental factors in developing psychosis [e.g., (31, 32)], and the need for mental healthcare to address themes such as social participation, resilience, and the establishment of supportive therapeutic relationships (33). However, since PR is - in part - distinct from symptomatic recovery in some ways, the interplay between affect, PR, and symptomatic remission from psychosis within the context of Soteria is an interesting subject for future research. Furthermore, ongoing research is looking into the longitudinal effects of Soteria on PR compared to care as usual.

Although this study focuses on Soteria and the way in which it aims at promoting PR, the five themes mentioned can be found in several other constructs. For instance, Hearing Voices Groups, in which group conversations with peers, within a non-judgmental atmosphere and a plurality of interpretations, are available, contribute to recovery (34). Resource groups acknowledge that promoting a person's recovery is a social process that takes place in relation to the social environment and everyday life (35). Finally, positive psychology approaches offered in a group intervention improve wellbeing by increasing self-acceptance and environmental mastery (36).

### 4.1. Limitations

Some limitations of the study are important to reflect on. First, the themes mainly discuss how Soteria facilitates PR, and they are less about hindering factors. The role of the first author as both researcher and former psychologist at Soteria could lead to the propensity to approach the data collection too optimistic. However, this was a constant subject of reflection during data collection and analysis, which was done by multiple people, who were not involved with the treatment at Soteria. Moreover, respondents were no longer treated at Soteria at the time of the interview, they were reassured that their answers had no consequences for current treatment, and hindering factors were specifically asked after. In qualitative research logic, results may be particularly hampered by reliance on a too-narrow sample that disables achieving a given research objective through data saturation. How we aimed to avoid this problem by doing the sampling purposefully was discussed in the method section. Nevertheless, we could have been even more focused on finding hindering factors, for instance, by specifically including patients that had left Soteria prematurely, a group that was not approached for participation in the current study based on exclusion criteria.

Second, the aim was not to conceptualize PR. However, in order to clarify what facilitated or hindered PR, it was necessary to ask participants how they defined PR. This led to variety in the meaning of PR. This raises the question of whether we really captured how to promote PR or mere recovery as a general construct. However, PR inherently is an idiosyncratic construct, and considering PR as “patient-based recovery” or recovery from the perspective of patients, the difference seems to fall off. Future research should be sensitive to these conceptual difficulties in assessing the patient perspective on factors promoting or hindering PR.

Finally, the generalizability of findings of a small sample qualitative study (like ours) is inherently limited. Indeed, the primary objective of such research is not a generalization, but typically in-depth exploration (in our study of factors that hinder and facilitate PR). What we did provide, however, as is common practice in qualitative research, are detailed descriptions of our findings and the context that we investigated. We hope that this enables readers to reflect on the extent to which and how our findings may also apply to other contexts and cases, and that it will provide sufficient input and direction for future (quantitative) studies on a larger scale.

### 4.2. Clinical implications

The overall aim of this study was to contribute to the understanding of how care settings may promote or hinder the process of PR in a population of people with psychotic disorders. Previous literature described what processes are involved with PR from psychosis. The current study gives a sense of how these processes can be put into practice. PR is a partly autonomous and idiosyncratic process, in which mental healthcare might have limited influence. However, by offering treatment within a normalizing, holding environment, with emphasis on equal and close contact, optimism and active structured days, and open-mindedness toward spirituality and the pros and cons of medication, PR can be facilitated without detracting from guideline-based treatment aimed at symptomatic recovery.

## Data availability statement

The raw data supporting the conclusions of this article will be made available by the authors, without undue reservation.

## Ethics statement

Ethical review and approval was not required for the study on human participants in accordance with the local legislation and institutional requirements. The patients/participants provided their written informed consent to participate in this study.

## Author contributions

PL conducted the interviews and analyzed the data in cooperation with the research team, consisting of M. Dieleman (MD) and A. Ruissen (AR). FH verified the analytical method and supervised the writing of the method and results section. All authors contributed to drafting the manuscript, the conception and design of the study, and approved the final version.
