# Supplementary material for: Facilitating and hindering factors of personal recovery in the context of Soteria—A qualitative study among people with (early episode) psychosis
Source: Front Psychiatry. 2023 Jan 4;13:1051446. doi: 10.3389/fpsyt.2022.1051446 (PMC9848445; doi:10.3389/fpsyt.2022.1051446)
Supplement: Supplementary file 1 [file Table_1.DOCX]

Appendix 1. Topic list final interview

| **Starting questions**  What does personal recovery mean to you?  (How) was attention paid to this at Soteria?  (How) was this helpful/hindering? | |
| --- | --- |
| **Topic based on literature** | **Question** |
| Peer support, connnectedness | Did spending time with others influence your PR?  (In which way) was attention given to this at Soteria?  (How) was this helpful/hindering in your PR? |
| Functioning | Did having structured days influence your PR?  (In which way) was attention given to this at Soteria?  (How) was this helpful/hindering in your PR? |
| Identity | Did going through psychosis influence your self-perception?  (In which way) was attention given to this at Soteria?  (How) was this helpful/hindering in your PR? |
| Hope and optimism | Was atmosphere of influence on your PR?  How would you describe the atmosphere at Soteria?  (How) did this help/ hinder your PR? |
| Relatives | Did relatives or other persons of reference influence you PR?  (In which way) was attention given to this at Soteria?  (How) was this helpful/hindering in your PR? |
| Spirituality | Did spirituality influence your PR?  (In which way) was attention given to this at Soteria?  (How) was this helpful/hindering in your PR? |
| Medication | Did medication influence your PR?  (In which way) was attention given to this at Soteria?  (How) was this helpful/hindering in your PR? |
| Strengths | How did your strenghts influence your PR  (In which way) was attention given to this at Soteria?  (How) was this helpful/hindering in your PR? |
| **Ending questions**  If Soteria would move to another place, what elements should they bring and what should they get rid of?  Is there anything we have not talked about that you would like to add? | |
